# Acute effects of different dynamic exercises on hamstring strain risk factors

**DOI:** 10.1371/journal.pone.0191801

**Published:** 2018-02-01

**Authors:** Che Hsiu Chen, Ye Xin, Kuang Wu Lee, Ming Ju Lin, Jiu Jenq Lin

**Affiliations:** 1 Department of Sport Performance, National Taiwan University of Sport, Taichung, Taiwan; 2 Graduate Institute of Sports Training University of Taipei, Taiwan; 3 Department of Health, Exercise Science, and Recreation Management, University of Mississippi, Mississippi, United States of America; 4 Department of Physical Education, Health and Recreation, National Chiayi University, 85 Wenlong Village, Minsyong Township, Chiayi County, Taiwan; 5 School and Graduate Institute of Physical Therapy, College of Medicine, National Taiwan University, Zhongzheng District, Taipei City, Taiwan; Nanyang Technological University, SINGAPORE

## Abstract

The purpose of the study was to examine the acute effects of different dynamic exercise interventions on hamstring muscle performance. Thirty-six young men with poor hamstring flexibility were randomly assigned to three intervention groups: jogging combined with dynamic open kinetic chain stretching (DS), jogging combined with dynamic closed kinetic chain stretching (lunge with eccentric hamstring windmills, LEC), and jogging only (CON) groups. Hamstring flexibility, muscle stiffness (area under the curve, AUC), joint position sense (JPS), maximal eccentric strength (ECC), and angle of peak torque (APT) were recorded before and immediately after the exercise interventions. The results showed that the hamstring flexibility increased in DS (*p* < 0.001); muscle stiffness decreased in DS and was lower than jogging (*p* < 0.001). Moreover, ECC increased in LEC and was higher than jogging and DS (*p* < 0.001). APT was different among 3 groups (*p* < 0.001). Decreased accuracy of JPS was found in DS and jogging (*p* < 0.001). In conclusion, the dynamic closed kinetic chain stretching (LEC) as compared to open kinetic chain stretching (DS) or jogging group, may be an effective technique to enhance muscle performance during the pre-competition warm-up routine.

## Introduction

Hamstrings muscle strain often occurs during sporting events/activities such as sprinting or kicking, where the muscle group is actively stretched by simultaneous hip flexion and knee extension movements [1]. In addition to inadequate hamstring muscle strength, altered knee kinematics, impaired knee joint proprioception, and poor hamstring flexibility are also primary risk factors for hamstrings strain [2, 3]. To prevent hamstring strain, traditional stretching exercise such as static, ballistic, or proprioceptive neuromuscular facilitation (PNF) stretching is recommended prior to exercise or sporting activities [4]. Generally speaking, these types of stretching interventions are believed to improve the range of motion (ROM) and to decrease the muscle stiffness, thereby serving as an important part of the warm-up procedure. The passive straight leg raise test is a common test to examine the hamstring muscle flexibility [5–7]. Thus, these stretching techniques may prevent risk factors of hamstring strain [3].

Muscle contraction during the lengthened or stretched state can alter force generation [8], the optimal sarcomere length [9], and the optimal joint angle [9]. For example, forward lunge with leg eccentric hamstring windmills exercise (LEC) is based on contraction by agonist muscle and stretching antagonist muscle simultaneously (put under tension by the lengthened muscle) [9]. Since muscle stiffness plays an important role for muscle performance and injury prevention [2], active contraction during stretching can be beneficial. Therefore, dynamic warmup intervention such as dynamic stretching, as opposed to static [10, 11] or PNF stretching [11], can serve as an effective technique for not only improving flexibility, but potentially enhancing lower extremity muscle performance [12, 13].

However, the stretching intensity, the volume, and the stretch velocity of dynamic stretching can impose differential effects on hamstrings performance [14]. For example, Herda et al.[15] reported that the active dynamic stretching of hamstrings (four sets of 12–15 repetitions with 20 seconds rest between sets, swinging the leg to the end range of motion and pulling the leg back toward body) resulted in an acute increase in passive ROM, but a decrease in isometric peak torque of the knee flexors. However, a different study demonstrated that two sets of 15 repetitions of squats on the floor has no influence on eccentric strength (60^0^/s and 180^0^/s), length-tension relationship, angle of peak torque, or total work [16]. In addition, the active dynamic stretching (two dynamic exercises with four sets of 30 seconds with 15 seconds rest between sets, including swinging the leg and squatting on the floor) led to decreased eccentric and concentric hamstring strength and hamstring-quadriceps strength ratio (60^0^/s and 180^0^/s) [17]. Therefore, a clear consensus for the effect of dynamic stretching on hamstring muscle performance has not been achieved, and the effects of different types of dynamic hamstring stretching exercises on hamstring muscle performance is still unclear.

To our knowledge, little information is known regarding the effects of using different dynamic hamstring stretching that involves swinging the leg into a stretched position (DS) versus forward lunge with single leg eccentric hamstring windmills (LEC) on hamstring strain risk factors (flexibility, eccentric strength, peak torque, muscle stiffness, knee proprioception). Therefore, the main purpose of this investigation was to examine the effects of three different dynamic exercises (jogging with DS vs. jogging with LEC vs. jogging only) on hamstring strain risk factors mentioned above. We expected to see that DS and LEC would exhibit significant increase in muscle performance and positive effects on risk factors than jogging group. In addition, due to the simultaneous activation of the shortened (quadriceps) and lengthened (hamstrings) muscles, LEC protocol may exhibit even greater treatment effects on muscle performance and greater positive effects on strain risk factors than DS protocol does.

## Materials and methods

### Participants

Thirty-six recreationally active yet untrained (i.e., not currently training in running, stretching and strength) male subjects (age 22.0 ± 1.5 yr, height 172.5 ± 2.9 cm, and weight 65.3 ± 6.4 kg) with poor hamstrings flexibility (< 80° passive straight leg raise) [18] voluntarily participated in this investigation. All subjects did not have a prior history of lower extremity injury or neurological disorder and low back pain prior to participate in this study. All subjects provided written informed consent before testing. This study was approved by the ethics committee on human research of National Taiwan University. The individual in this manuscript has given written informed consent (as outlined in PLOS consent form) to publish these case details.

### Experimental design and procedures

**Fig 1:**
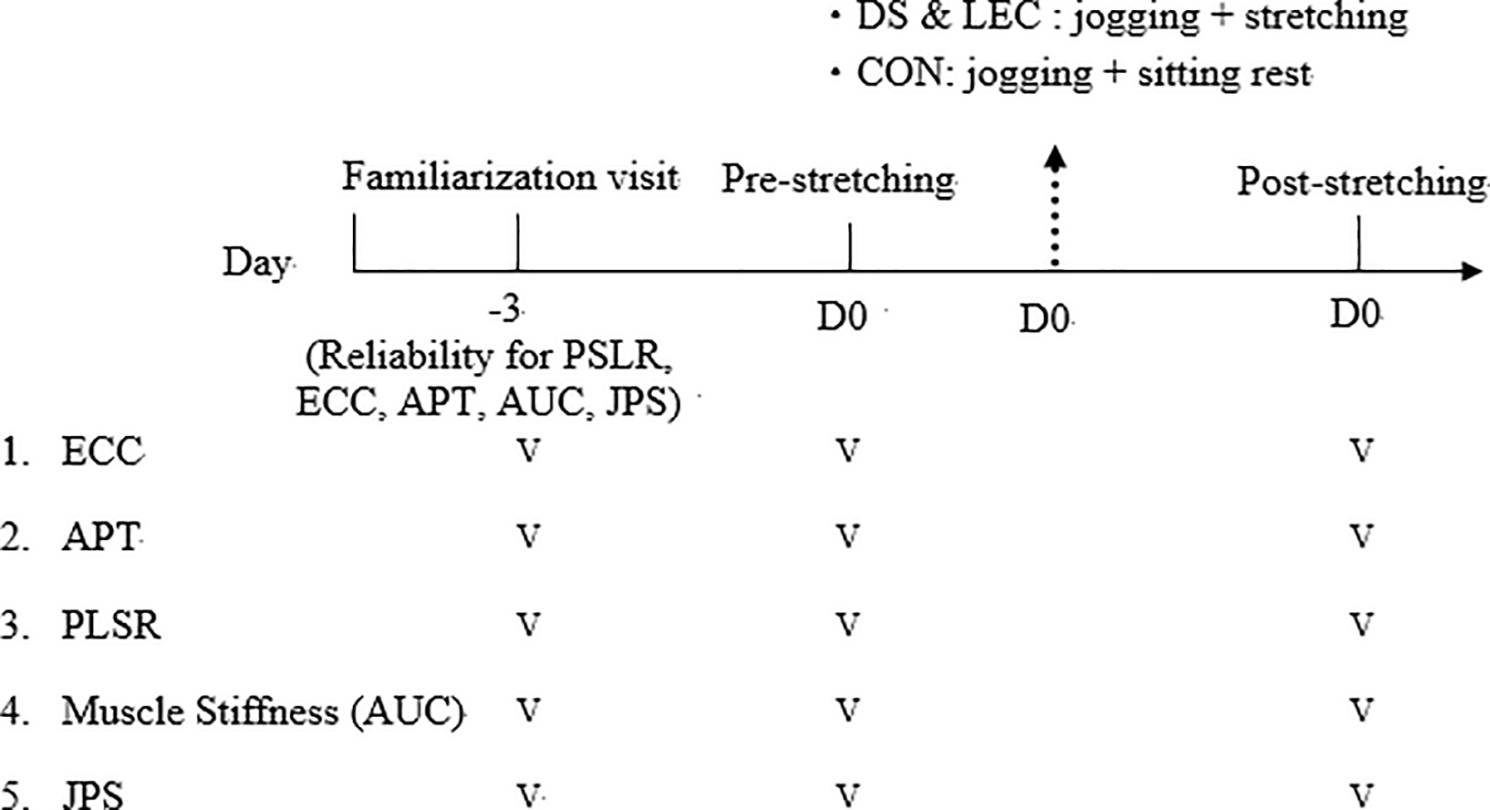
Schematic representation of experimental design. D0: Testing day (Experimental Visit).

The study was randomized control trial. This investigation used a between-group design to examine the acute effects of three dynamic exercise interventions (jogging with DS vs. jogging with LEC vs. jogging only) on hamstring performance and strain risk factors. The subjects were randomly divided into three groups (n = 12 per group): jogging only (CON), jogging with LEC, and jogging with DS. All testing was performed on the dominant side of the hamstring muscles. Three days prior to Experimental Visit, all subjects participated in an introductory session (Familiarization Visit) during which they were familiarized with the designated exercise intervention and the testing procedures. Before (Pre) and immediately after (Post) the exercise intervention, the maximal voluntary eccentric strength (ECC), angle of peak torque (APT), muscle flexibility (passive straight leg raise test: PSLR), muscle stiffness (area under the curve, AUC), and joint position sense (JPS) were measured (see in Fig 1).

#### Dynamic exercise interventions

**Fig 2:**
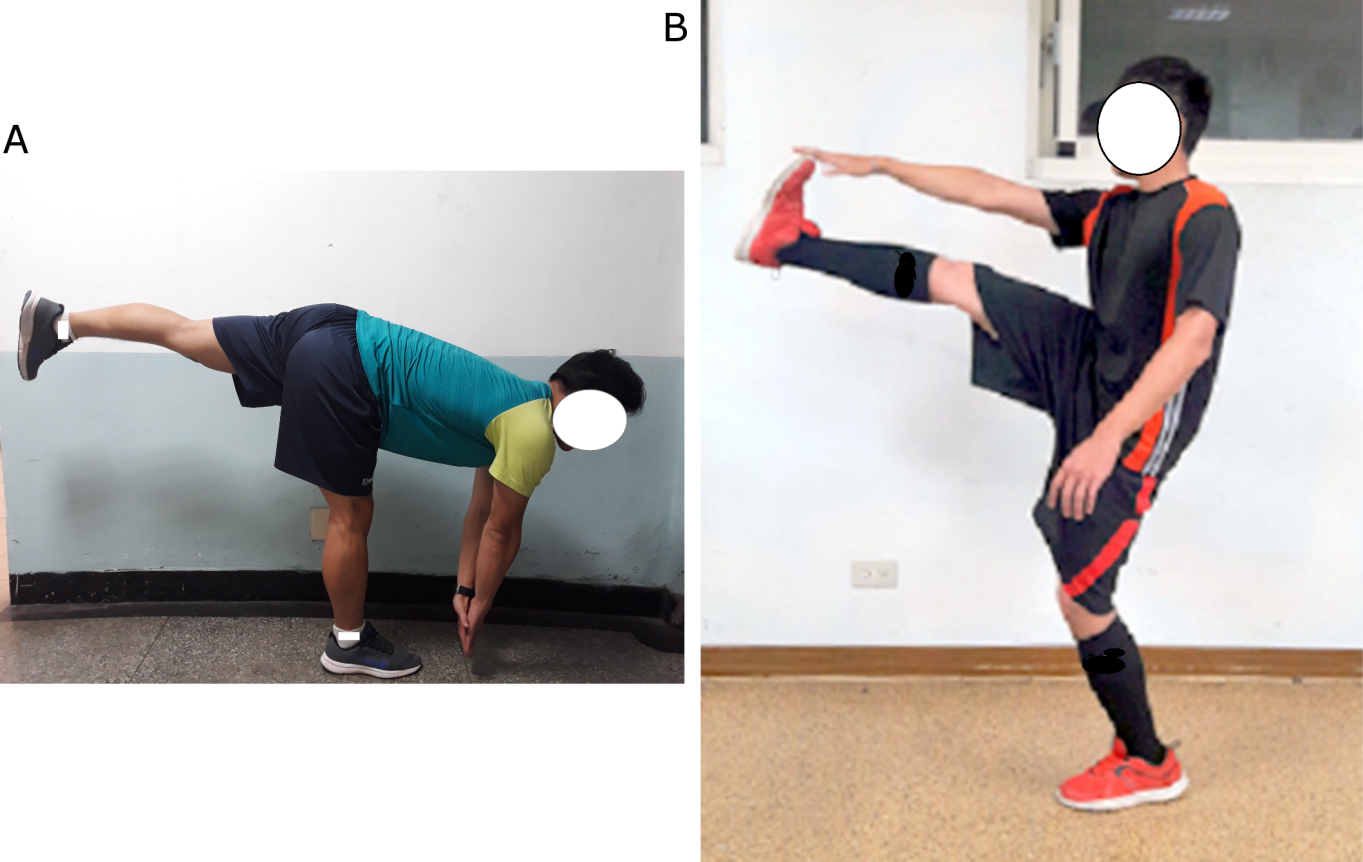
The two types of dynamic stretching exercises for the hamstring muscles. (A) Dynamic closed kinetic chain stretching (LEC). (B) Dynamic open kinetic chain stretching (DS).

Upon arrival during the Experimental Visit, all subjects began with a jogging for 5 minutes on a treadmill at 6.4 km·h^-1^ with 1% grade. Following this light warm-up exercise, the subjects were asked to perform the following designated interventions:

Jogging with LEC (LEC). The LEC is performed as the closed kinetic chain exercise intervention. First, the subject was instructed to take a dominant leg to the stepping forward position with the dominant knee slight bend (10–15°) [18]. Then, the subject slowly bent the upper body forward until reaching the end of the hip flexion range of motion. The knee was extended simultaneously to stretch the hamstring muscles. The intensity of stretching was set at less than the point of discomfort [19]. With one second down and one second up back to the starting position, six 15-second LEC intervention were performed with 15 seconds of rest between consecutive sets. Therefore, a total of 48 repetitions (6 sets × 8 repetitions per set) of LEC were performed (Fig 2A).

Jogging with DS (DS). The DS exercise is performed as the open kinetic chain exercise intervention. First, the subject was instructed to raise the arm horizontal to the floor with the pelvic tilting anteriorly in the standing position. Second, the subject actively swang the dominant leg forward to the end of the hip flexion range of motion. During this exercise, the knee was kept extended and the subject was allowed to use the hand to approach the foot. The intensity of stretching was set at less than the point of discomfort. The leg was then swung back slightly passing the starting position. Instruction to the subject was emphasized so the upper body was always kept straight. Similar to the LEC intervention, 6 sets of 15-second DS were performed with a rest period of 15 seconds between sets. Both LEC and DS stretching exercises were rhythmic movements and they were set at a rhythm of 60 beats/min by a metronome (Seiko, DM70 Digital Metronome, China) (Fig 2B).

The jogging only group (CON) did not perform any other interventions, instead 5 additional minutes of jogging (same speed and inclination) were performed.

#### Performance testing

Testing order for dependent variables was muscle flexibility, muscle stiffness, joint position sense, maximal voluntary eccentric strength, and angle of peak torque:

Hamstring flexibility (passive straight leg raise, PSLR). The subject lay supine on a padded table and both waist and the non-stretched leg were fixed by a strap. The first examiner held the subject’s dominant leg and moved the leg to the position when the subject felt a mild sensation of pain, and the digital inclinometer (inclinometer, Model # A800; JIN-BOMB Inc, Kaohsiung, Taiwan) was placed over the distal tibia. The non-stretched leg was fully extended by a strap, and the second examiner held the pelvis to avoid posterior rotation. This test was repeated 3 times, and the average of 3 measures was used for analysis [3, 20].

Muscle stiffness (area under the curve, AUC). Hamstrings muscle stiffness was quantified by a Myotonometer (Neurogenic Technologies, Inc, Missoula, MT), a computerized meter-type device to measure relaxed muscle-stiffness levels. The Myotonometer has been demonstrated to be valid and reliable to measure muscle stiffness [21]. The head of the Myotonometer probe was placed along the longitudinal axis of the dominant leg’s biceps femoris muscle at 50% of the distance from the ischial tuberosity to the medial epicondyle of the tibia. The tissue-displacement was made at 8 force increments of probe pressure (0.25, 0.50, 0.75, 1.00, 1.25, 1.50, 1.75, 2.00 kg) and computational software creates force displacement curves (AUC, mm/kg) based on these data. A more compliant (lower stiffness, higher AUC) muscle has a sharper slope of the force-displacement curve than a muscle with higher stiffness (lower AUC) [20, 21].

Joint position sense (JPS). The JPS assessment involved an active positioning and repositioning (active test) of the dominant leg. The measurements were taken at an isokinetic dynamometer (Biodex Medical Systems, Inc., Shirley, NY, USA) with the subject lying in the prone position. All subjects had the “hold” button in one hand so that they could stop the lever arm of the dynamometer by pressing button when they reached target angle and held it about 2 seconds [22]. The starting position was at the knee full extension and subjects actively moved their limbs to the target angle (30^0^, 50^0^ and 70^0^ of knee flexion). In each trial, the lower leg was passively moved to target position at slow angular velocity (10^0^/s) while maintaining the target position for 10 seconds. The protocol of the JPS assessment was completed by the same researcher. After learning and practice, subjects were then blindfolded and actively moved their leg to the target positions. This procedure was repeated five times for each testing positions. The repositioning absolute angular error (AAE) was obtained through the calculation of the difference between the target angle and repositioning angle [23].

Isokinetic strength testing. The maximal isokinetic hamstrings eccentric peak torque (ECC) testing was performed on the same isokinetic dynamometer as the JPS was measured. The subject lay prone on the platform of the dynamometer, with upper back, lower back and contralateral leg strapped to the platform. The lateral condyle of the femur was aligned with the rotation axis of the dynamometer. The pad of the dynamometer’s lever arm was secured around the ankle and the base of the pad was approximately 5 cm proximal to the malleoli. The knee range of motion (ROM) was set at 0^0^ (full extended position) to 110^0^ of flexion. Three submaximal warm-up trials (50% of self-perceived effort) preceded three maximal isokinetic eccentric muscle contractions at 30^0^/s with a 45 seconds rest between each muscle contraction. During the test, strong verbal encouragement was provided during contractions. The joint angle of peak torque (APT) was provided by Biodex System software.

#### Statistical analyses

A priori power analyses (G*Power 3.1) indicated that a sample size of 10 subjects per group would result in statistical power values of 0.80 or greater for all the dependent variables [24]. The Shapiro–Wilk test was to confirm normality of the data. Separate two-way mixed analyses of variance (ANOVAs) (experimental group [DS vs. LEC vs. CON] × time [Pre vs. Post]) were used for each dependent variable. When appropriate, follow-up tests included one-way ANOVAs and pairwise comparisons. Effect sizes Cohen’s d (ES) were calculated by: Cohen’s *d* = *Mean*_1_—*Mean*_2_ / SD_pooled_, where SD_pooled_ = √[(SD_1_^2^+ SD_2_^2^) / 2] [25]. Specifically, *d* = 0.2 can be considered a 'small' effect size, 0.5 represents a 'medium' effect size and 0.8 a 'large' effect size. To determine the test–retest reliability of the outcome measures, intraclass correlation coefficient (ICC) scores for PSLR, ECC, APT, AUC, JPS (30^0^), JPS (50^0^), and JPS (70^0^) were calculated for values measured during the Familiarization Visit and the Pre-intervention during the Experimental Visit. All statistical analyses were conducted using SPSS 17.0 with an alpha level of .05.

## Results

The mean values and SEM of the different variables assessed are shown in Table 1. Before the dynamic exercise intervention, there is no significant difference in all dependent variables among all three groups. Normality of the data was also confirmed. The ICCs for all dependent variables were between 0.80–0.93 with adequate reliability.

**Table 1:** Pre vs. Post in outcomes (mean ± SEM) before and after three dynamic warm-up exercises.

	Pre-Stretching			Post-Stretching		
Variable	CON	LEC	DS	CON	LEC	DS
PSLR (^0^)	41.9±2.4	40.0±2.9	38.6±2.7	37.5±2.3^*^	46.1±2.8^*^^#^	50.4±2.4^*^^#^
AUC (mm/kg)	17.6±0.7	17.9±0.5	17.9±0.5	16.2±0.6^*^	18.2±0.5^+^	19.0±0.5^*^^#^
ECC (Nm)	76.7±6.4	82.1±5.0	80.1±6.2	76.1±6.4	92.6±6.1^*^^#^^+^	65.7±4.6^*^
APT (^0^)	29.7±1.3	26.7±2.0	26.7±1.7	27.2±0.9^*^^+^	24.1±1.5^*^^#^^+^	34.5±1.6^*^^#^
JPS (30^0^)	7.3±1.9	5.8±1.5	5.0±1.5	9.8±1.3^*^	4.5±0.8^#^^+^	9.2±1.1^*^
JPS (50^0^)	2.5±0.4	3.7±0.7	3.3±0.8	7.3±1.1^*^	3.7±0.6^#^^+^	6.8±0.5^*^
JPS (70^0^)	3.4±0.3	3.1±0.5	3.3±0.2	4.6±0.1^*^	3.0±0.5^#^^+^	5.3±0.3^*^

CON: jogging only; DS: dynamic open kinetic chain stretch; LEC: dynamic closed kinetic chain stretching; PSLR: passive straight-leg raising; AUC: area under the curve; ECC: maximal eccentric isokinetic strength; APT: angle of peak torque; JPS: joint position sense

* Significant (P< 0.05) difference from the Pre-value.

# Significant (P< 0.05) difference from the CON group.

+ Significant (P< 0.05) difference from the DS group.

Significant 2-way group by time interactions were found for all outcomes (*P* < 0.001). There was a significant group by time interaction on PSLR (*F*_2, 33_ = 114.29, *P* < 0.001, partial eta squared = 0.88). PSLR increased significantly in DS and LEC (*P* < 0.001, effect size = 0.94 and 0.99), but decreased significantly in CON (*P* = 0.04, effect size = 0.56). In addition, the DS exercise resulted in significant greater PSLR increments than the LEC did (*P* = 0.01) (Fig 4A).

**Fig 3:**
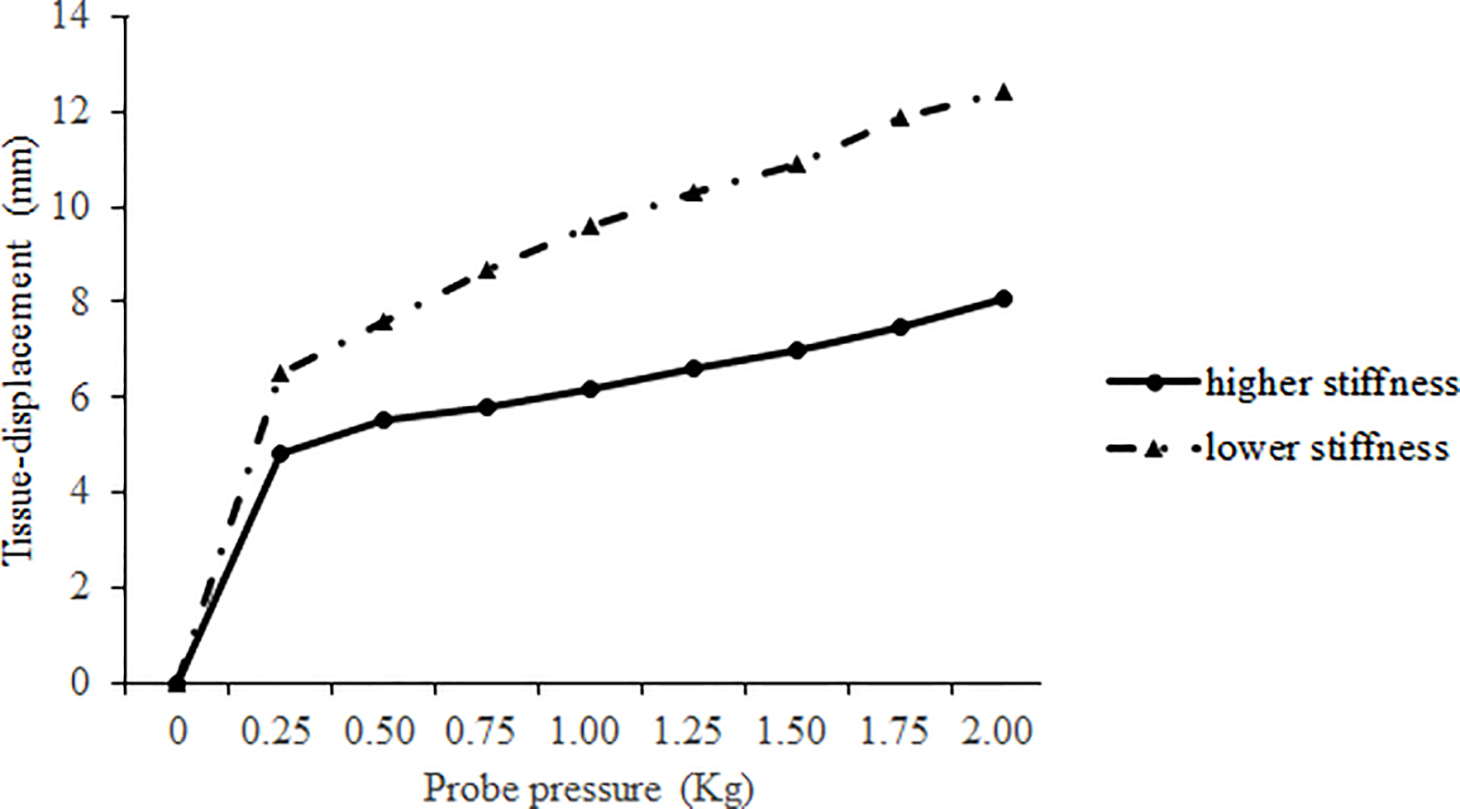
An example of using AUC (area under the curve) for the assessment of muscle stiffness.

Fig 3 shows a typical example of muscle stiffness by the measurement of AUC. There was a significant group by time interactions on AUC (*F*_2, 33_ = 25.31, *P* < 0.001, partial eta squared = 0.61). AUC decreased significantly in CON (*P* < 0.001, effect size = 0.99) and increased significantly in DS (*P* = 0.01, effect size = 0.53). There was no significant AUC change in LEC (*P* > 0.05, effect size = 0.004). In addition, there is a significant difference between the AUC values of Post-DS and Post-CON (*P* < 0.001).

As for the JPS, there were significant group by time interaction at different angles (30^0^: *F*_2, 33_ = 15.10, *P* < 0.001, partial eta squared = 0.48; 50^0^: *F*_2, 33_ = 9.86, *P* < 0.001, partial eta squared = 0.37; 70^0^: *F*_2, 33_ = 4.10, *P* = 0.03, partial eta squared = 0.20). The JPS (30^0^) error increased significantly in CON (30^0^: *P* < 0.001, effect size = 0.44; 50^0^: *P* < 0.001, effect size = 0.62; 70^0^: *P* < 0.001, effect size = 0.70) and DS (30^0^: *P* < 0.001, effect size = 0.89; 50^0^: *P* < 0.001, effect size = 0.80; 70^0^: *P* < 0.001, effect size = 0.80). There was no significant difference in JPS error for LEC group. In addition, the JPS errors were also significantly larger in CON and DS groups when compared with the LEC (Table 1).

**Fig 4:**
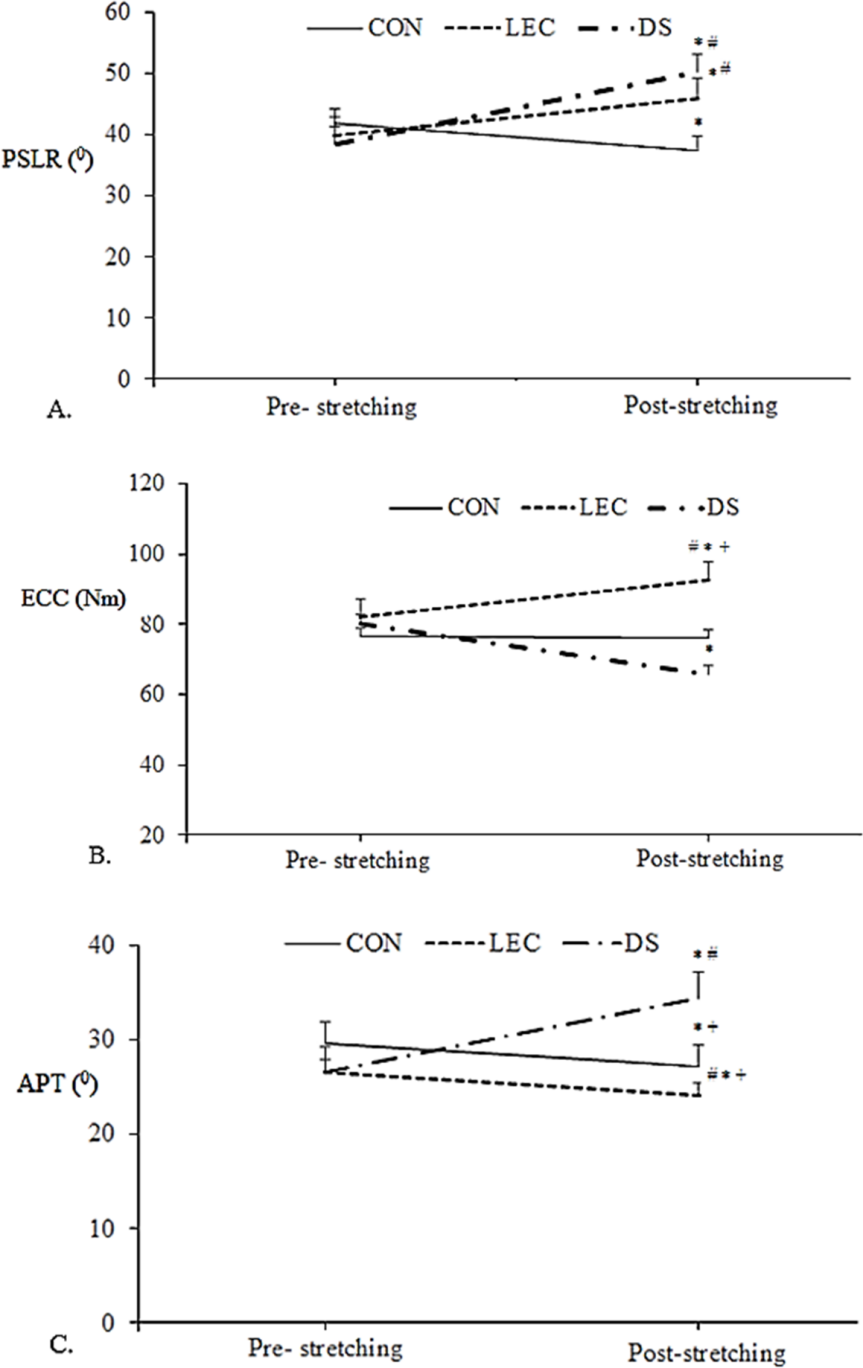
Measurements (mean ± SEM) before and after three dynamic stretching exercises. (A) The passive straight-leg raising (PSLR). (B) Maximal eccentric isokinetic strength (ECC). (C) Angle of peak torque (APT). CON: jogging only; DS: dynamic open kinetic chain stretching; LEC: dynamic closed kinetic chain stretching. * Significant (P< 0.05) difference from the Pre-value. # Significant (P< 0.05) difference from the CON group. + Significant (P< 0.05) difference from the DS group.

As for the eccentric strength, there was a significant group by time interaction on (*F*_2, 33_ = 10.23, *P* < 0.001, partial eta squared = 0.38). ECC decreased significantly in DS (*P* < 0.001, effect size = 0.43) and increased significantly in LEC (*P* = 0.02, effect size = 0.80). ECC was also significantly higher in LEC when compared with DS and CON after the intervention (Fig 4B).

Lastly, the two-way mixed factorial ANOVA showed that there was a significant group by time interaction for APT (*F*_2, 33_ = 49.99, *P* < 0.001, partial eta squared = 0.75). After the interventions, the APT in DS was significantly larger than that in LEC (*P* < 0.001, effect size = 0.52) and in CON (*P* < 0.001, effect size = 0.35). The APT in LEC was significantly lower than that in CON (*P* < 0.001). In addition, the results also showed that after the interventions, the APT shifted to the shorter muscle length in DS group (*P* < 0.001) but to the longer muscle length in LEC (*P* < 0.001) and in CON (*P* = 0.03) (Fig 4C).

## Discussion

The present study evaluated the acute effects of three dynamic exercise interventions (jogging only vs. jogging with DS vs jogging with LEC) on hamstring strain risk factors (flexibility, muscle stiffness, muscle strength, angular peak torque, and joint position senses).

Our findings showed that there is an increase in ROM following DS and LEC. These results are in agreement with previous studies [12, 14]. For example, similar to a previous study [26], increases in ROM occurred after LEC (27.9%) and DS (12.4%). However, AUC did not have similar responses with muscle flexibility, with DS decreasing the muscle stiffness, but LEC having no effect. These findings are in agreement with the results of previous studies that stretching can improve hamstring ROM but not necessarily by improving the muscle compliance [20]. This may indicate that the stretching techniques increase joint ROM as a result of a change in stretch tolerance rather than the passive properties of muscle [27].

When comparing two dynamic stretching protocols, DS impaired hamstring muscle performance (decreased eccentric strength). This result is different from what have been reported from previous research, including the one from Sekir et al. [7], where the authors reported that DS improved the muscular strength. It should be noted that the difference in muscular performance following dynamic stretching can be due to the slightly different stretching interventions used. For example, Sekir et al. [7] had subjects perform the DS with both hamstring concentric contraction (knee flexion) and eccentric contraction (knee extension) phases combined. However, our DS intervention only focused on the portion of hamstring eccentric contraction. Therefore, the eccentric-only exercise might have impaired the subsequent muscular strength performance. In addition to the strength performance, the APT shifts to the shorter muscle length following DS, which suggests that the increased muscle compliance can be related to decrease in sarcomere connection at longer muscle length position. On the other hand, the LEC shifted the APT value to the longer muscle lengths and reduced the risk of hamstring injury during eccentric contraction. Our results demonstrate that decreased eccentric peak torque (-17.8%) occurs following DS while increased eccentric peak torque (12.8%) takes place following LEC.

Using DS or LEC for improving joint proprioception is not likely to occur. Researchers have examined the effect of warm-ups consisting of jogging and stretching exercise or only stretching exercise on knee joint proprioception [22, 28, 29]. Knee joint proprioception did not change after stretching and one study demonstrated that the PNF increases knee movement errors [28]. Similarly, our study found no improvement on proprioception in LEC and decreased proprioception of knee movements in both the jogging and DS groups. It has been speculated that stretching of the hamstring and quadriceps may reduce muscle stiffness [28] and reduce sensitivity of the muscle spindle activity [30]. Specifically, the acute decrease in muscle strength following stretching could apparently affect proprioceptive sensitivity of the muscle [31]. The decreased eccentric peak torque and increased knee JPS error (300, 50^0^ and 70^0^ of flexion) of DS group in our finding support this assumption. This may also be susceptible to injury of knees [29]. On the other hand, LEC seems to prevent this negative effect of stretching on joint proprioception in our findings.

Muscle performance after stretching can be associated with mechanical factors (decrease in muscle stiffness or change in the length-tension relationship) or neural factors (decrease in motor unit activation or post activation potentiation) [12, 28]. Based on our finding on the increased ECC strength, the LEC enhanced neuromuscular performance of the stretched muscle. Further, LEC does not impair knee joint proprioception. Similarly, researchers found that the dynamic warm-up exercise with weight bearing as LEC can improve jump performance and propose increase in post activation potentiation during DS [32].

Limitation of this study should be noted. First and foremost, although we directly compared DS and LEC interventions, it was impossible to perfectly match the exercise volume between both interventions. Based on our observation, for every stretch, the LEC condition appeared to provide a longer time for the hamstring muscle under stretch than DS did. And the subjects had to focus more to control the posture during the LEC rather than the DS. Thus, these differences might have contributed the subsequent muscle performance differences. Second, the subjects investigated in this study were only untrained male subjects, whether the DS and LEC exercises affect the genders or subject training experience and background is not clear. Although significant changes in the angle of peak torque in DS (29.2%) and LEC (9.7%) and the significant increase in PSLR for LEC (15.3%) and DS (30.6%) immediately after warm-up, long term influence of DS combined with LEC exercises on the hamstring muscle should be further investigated.

## Conclusion

The short stretching duration (90 seconds) in dynamic closed kinetic chain stretching (LEC) can improve muscle performance. Although increased flexibility and muscle compliance can be found after open kinetic chain stretching (DS), it may impair eccentric strength, change the angle of peak torque and diminish knee joint proprioception sensation. Therefore, the LEC exercise is recommended as a warm-up protocol.

## References

[1] Witvrouw E, Danneels L, Asselman P, D'Have T, Cambier D. Muscle flexibility as a risk factor for developing muscle injuries in male professional soccer players. A prospective study. Am J Sports Med. 2003;31(1):41–6. doi: 10.1177/03635465030310011801 . 12531755 10.1177/03635465030310011801

[2] Watsford ML, Murphy AJ, McLachlan KA, Bryant AL, Cameron ML, Crossley KM, et al A prospective study of the relationship between lower body stiffness and hamstring injury in professional Australian rules footballers. Am J Sports Med. 2010;38(10):2058–64. doi: 10.1177/0363546510370197 . 20595555 10.1177/0363546510370197

[3] Chen CH, Nosaka K, Chen HL, Lin MJ, Tseng KW, Chen TC. Effects of flexibility training on eccentric exercise-induced muscle damage. Med Sci Sports Exerc. 2011;43(3):491–500. doi: 10.1249/MSS.0b013e3181f315ad . 20689450 10.1249/MSS.0b013e3181f315ad

[4] Thacker SB, Gilchrist J, Stroup DF, Kimsey CD Jr. The impact of stretching on sports injury risk: a systematic review of the literature. Med Sci Sports Exerc. 2004;36(3):371–8. . 15076777 10.1249/01.mss.0000117134.83018.f7

[5] Lopez-Minarro PA, Muyor JM, Belmonte F, Alacid F. Acute effects of hamstring stretching on sagittal spinal curvatures and pelvic tilt. J Hum Kinet. 2012;31:69–78. doi: 10.2478/v10078-012-0007-7 . 23486214 10.2478/v10078-012-0007-7 PMC3588653

[6] Muyor JM, Alacid F, Lopez-Minarro PA. Influence of hamstring muscles extensibility on spinal curvatures and pelvic tilt in highly trained cyclists. J Hum Kinet. 2011;29:15–23. doi: 10.2478/v10078-011-0035-8 . 23486997 10.2478/v10078-011-0035-8 PMC3588616

[7] Muyor JM, Arrabal-Campos FM. Effects of Acute Fatigue of the Hip Flexor Muscles on Hamstring Muscle Extensibility. J Hum Kinet. 2016;53:23–31. doi: 10.1515/hukin-2016-0007 . 28149407 10.1515/hukin-2016-0007 PMC5260573

[8] Gleim GW, McHugh MP. Flexibility and its effects on sports injury and performance. Sports Med. 1997;24(5):289–99. . 9368275 10.2165/00007256-199724050-00001

[9] Pacheco L, Balius R, Aliste L, Pujol M, Pedret C. The acute effects of different stretching exercises on jump performance. J Strength Cond Res. 2011;25(11):2991–8. doi: 10.1519/JSC.0b013e318212dac0 . 21993032 10.1519/JSC.0b013e318212dac0

[10] Sekir U, Arabaci R, Akova B, Kadagan SM. Acute effects of static and dynamic stretching on leg flexor and extensor isokinetic strength in elite women athletes. Scand J Med Sci Sports. 2010;20(2):268–81. doi: 10.1111/j.1600-0838.2009.00923.x . 19486475 10.1111/j.1600-0838.2009.00923.x

[11] Manoel ME, Harris-Love MO, Danoff JV, Miller TA. Acute effects of static, dynamic, and proprioceptive neuromuscular facilitation stretching on muscle power in women. J Strength Cond Res. 2008;22(5):1528–34. doi: 10.1519/JSC.0b013e31817b0433 . 18714235 10.1519/JSC.0b013e31817b0433

[12] Fletcher IM, Anness R. The acute effects of combined static and dynamic stretch protocols on fifty-meter sprint performance in track-and-field athletes. J Strength Cond Res. 2007;21(3):784–7. doi: 10.1519/R-19475.1 . 17685686 10.1519/R-19475.1

[13] Behm DG, Chaouachi A. A review of the acute effects of static and dynamic stretching on performance. Eur J Appl Physiol. 2011;111(11):2633–51. doi: 10.1007/s00421-011-1879-2 . 21373870 10.1007/s00421-011-1879-2

[14] Fletcher IM. The effect of different dynamic stretch velocities on jump performance. Eur J Appl Physiol. 2010;109(3):491–8. doi: 10.1007/s00421-010-1386-x . 20162300 10.1007/s00421-010-1386-x

[15] Herda TJ, Herda ND, Costa PB, Walter-Herda AA, Valdez AM, Cramer JT. The effects of dynamic stretching on the passive properties of the muscle-tendon unit. J Sports Sci. 2013;31(5):479–87. doi: 10.1080/02640414.2012.736632 . 23113555 10.1080/02640414.2012.736632

[16] Ayala F, De Ste Croix M, Sainz De Baranda P, Santonja F. Acute effects of static and dynamic stretching on hamstring eccentric isokinetic strength and unilateral hamstring to quadriceps strength ratios. J Sports Sci. 2013;31(8):831–9. doi: 10.1080/02640414.2012.751119 . 23230900 10.1080/02640414.2012.751119

[17] Costa PB, Herda TJ, Herda AA, Cramer JT. Effects of dynamic stretching on strength, muscle imbalance, and muscle activation. Med Sci Sports Exerc. 2014;46(3):586–93. doi: 10.1249/MSS.0000000000000138 . 24042312 10.1249/MSS.0000000000000138

[18] Sullivan MK, Dejulia JJ, Worrell TW. Effect of pelvic position and stretching method on hamstring muscle flexibility. Med Sci Sports Exerc. 1992;24(12):1383–9. . 1470022

[19] Batista LH, Vilar AC, de Almeida Ferreira JJ, Rebelatto JR, Salvini TF. Active stretching improves flexibility, joint torque, and functional mobility in older women. Am J Phys Med Rehabil. 2009;88(10):815–22. doi: 10.1097/PHM.0b013e3181b72149 . 21119314 10.1097/PHM.0b013e3181b72149

[20] Chen CH, Huang TS, Chai HM, Jan MH, Lin JJ. Two stretching treatments for the hamstrings: proprioceptive neuromuscular facilitation versus kinesio taping. J sport Rehabil. 2013;22(1):59–66. . 23069636 10.1123/jsr.22.1.59

[21] Aarrestad DD, Williams MD, Fehrer SC, Mikhailenok E, Leonard CT. Intra- and interrater reliabilities of the Myotonometer when assessing the spastic condition of children with cerebral palsy. J Child Neurol. 2004;19(11):894–901. doi: 10.1177/08830738040190110801 . 15658795 10.1177/08830738040190110801

[22] Larsen R, Lund H, Christensen R, Rogind H, Danneskiold-Samsoe B, Bliddal H. Effect of static stretching of quadriceps and hamstring muscles on knee joint position sense. Br J Sports Med. 2005;39(1):43–6. doi: 10.1136/bjsm.2003.011056 . 15618341 10.1136/bjsm.2003.011056 PMC1725022

[23] Torres R, Vasques J, Duarte JA, Cabri JM. Knee proprioception after exercise-induced muscle damage. Int J Sports Med. 2010;31(6):410–5. doi: 10.1055/s-0030-1248285 . 20301043 10.1055/s-0030-1248285

[24] Faul F EE, Lang AG, Buchner A. G*Power 3: a flexible statistical power analysis program for the social, behavioral, and biomedical sciences. Behav Res Methods. 2007;39(2):175–91. 17695343 10.3758/bf03193146

[25] J C. Statistical Power Analysis for the Behavioral Sciences. New York, NY: Routledge Academic 1988.

[26] Behm DG, Plewe S, Grage P, Rabbani A, Beigi HT, Byrne JM, et al Relative static stretch-induced impairments and dynamic stretch-induced enhancements are similar in young and middle-aged men. Appl Physiol Nutr Metab. 2011;36(6):790–7. doi: 10.1139/h11-107 . 22014144 10.1139/h11-107

[27] Magnusson SP. Passive properties of human skeletal muscle during stretch maneuvers. A review. Scand J Med Sci Sports. 1998;8(2):65–77. . 9564710 10.1111/j.1600-0838.1998.tb00171.x

[28] Streepey JW, Mock MJ, Riskowski JL, Vanwye WR, Vitvitskiy BM, Mikesky AE. Effects of quadriceps and hamstrings proprioceptive neuromuscular facilitation stretching on knee movement sensation. J Strength Cond Res. 2010;24(4):1037–42. doi: 10.1519/JSC.0b013e3181d09e87 . 20300021 10.1519/JSC.0b013e3181d09e87

[29] Bartlett MJ, Warren PJ. Effect of warming up on knee proprioception before sporting activity. Br J Sports Med. 2002;36(2):132–4. doi: 10.1136/bjsm.36.2.132 . 11916897 10.1136/bjsm.36.2.132 PMC1724483

[30] Avela J, Kyrolainen H, Komi PV. Altered reflex sensitivity after repeated and prolonged passive muscle stretching. J Appl Physiol. 1999;86(4):1283–91. doi: 10.1152/jappl.1999.86.4.1283 . 10194214 10.1152/jappl.1999.86.4.1283

[31] Bjorklund M, Djupsjobacka M, Crenshaw AG. Acute muscle stretching and shoulder position sense. J Athl Train. 2006;41(3):270–4. . 17043694 PMC1569556

[32] Thompsen AG, Kackley T, Palumbo MA, Faigenbaum AD. Acute effects of different warm-up protocols with and without a weighted vest on jumping performance in athletic women. J Strength Cond Res. 2007;21(1):52–6. doi: 10.1519/R-18965.1 . 17313270 10.1519/00124278-200702000-00010

